# Gut microbiota derived trimethylamine N-oxide (TMAO) detection through molecularly imprinted polymer based sensor

**DOI:** 10.1038/s41598-020-80122-6

**Published:** 2021-01-14

**Authors:** G. B. V. S. Lakshmi, Amit K. Yadav, Neha Mehlawat, Rekha Jalandra, Pratima R. Solanki, Anil Kumar

**Affiliations:** 1 Special Center for Nanoscience, Jawaharlal Nehru University, New Delhi, India; 2 Amity Institute of Applied Sciences, Amity University, Uttar Pradesh, Noida, India; 3 Department of Zoology, Maharshi Dayanand University, Rohtak, 124001 India; 4 National Institute of Immunology, New Delhi, India

**Keywords:** Biotechnology, Cancer, Biomarkers, Diseases, Health occupations, Medical research, Engineering, Materials science, Nanoscience and technology

## Abstract

Trimethylamine N-oxide (TMAO), a microbiota-derived metabolite has been implicated in human health and disease. Its early detection in body fluids has been presumed to be significant in understanding the pathogenesis and treatment of many diseases. Hence, the development of reliable and rapid technologies for TMAO detection may augment our understanding of pathogenesis and diagnosis of diseases that TMAO has implicated. The present work is the first report on the development of a molecularly imprinted polymer (MIP) based electrochemical sensor for sensitive and selective detection of TMAO in body fluids. The MIP developed was based on the polypyrrole (PPy), which was synthesized via chemical oxidation polymerization method, with and without the presence of TMAO. The MIP, NIP and the non-sonicated polymer (PPy-TMAO) were separately deposited electrophoretically onto the hydrolyzed indium tin oxide (ITO) coated glasses. The chemical, morphological, and electrochemical behavior of MIP, non-imprinted polymer (NIP), and PPy-TMAO were characterized using Fourier transform infrared spectroscopy (FT-IR), scanning electron microscopy (SEM), and electrochemical techniques. The detection response was recorded using differential pulse voltammetry (DPV), which revealed a decrease in the peak current with the increase in concentration of TMAO. The MIP sensor showed a dynamic detection range of 1–15 ppm with a sensitivity of 2.47 µA mL ppm^−1^ cm^−2^. The developed sensor is easy to construct and operate and is also highly selective to detect TMAO in body fluids such as urine. The present research provides a basis for innovative strategies to develop sensors based on MIP to detect other metabolites derived from gut microbiota that are implicated in human health and diseases.

## Introduction

There are increasing shreds of evidence that Trimethylamine-N-oxide (TMAO) is among the critical metabolites of gut bacteria. Research reports have found its association with adverse cardiac events and chronic kidney diseases^1,2^. Choline and choline containing compounds present in the dietary food are metabolized by gut bacteria to give rise Trimethylamine (TMA), the precursor of TMAO. The flavin-dependent monooxygenase (FMO) isoforms 1 and 3 are responsible for converting TMA to TMAO in the liver^3^. Recent research progress on TMAO suggests that TMAO can be a diagnostic and prognostic marker for colorectal cancer (CRC), cardiovascular, diabetes and other diseases^4,5^. Suzuki et al.^6^ suggested that TMAO can be used in secondary risk stratification for patients suffering from acute coronary syndrome. Another study found that TMAO may be considered an early marker of atherosclerosis^7^. TMAO may also be regarded as a prognostic marker in pneumonia, as proved by a recent study carried out by Ottiger et al.^8^. Various reports are coming up to use TMAO as prognostic and diagnostic markers, which delineate its biological functions in human health and diseases.

Previously, gas chromatography, high-performance liquid chromatography (HPLC), nuclear magnetic resonance (NMR), and mass spectrometry-based methods have been used for the detection of TMA and TMAO^9–11^. Biljanamitrova et al., described the first enzyme-based biosensor for TMAO detection, having a linear range of 2–110 mM, limit of detection of (LOD) 2.96 nM with a sensitivity of 14.16 nA/μM^12^. Also, Sunil Veeravalli et al. developed a method for TMA, TMAO, and creatinine detection in mouse urine having LOD of 115 pg/mL with the measured linearity of the calibration curves of 15–944 pg/μL^13^. Qiuwu et al. developed a rapid ultra performance liquid chromatography-mass specroscopy (UPLC-MS/MS) method for simultaneous detection of TMAO, TMA, and dimethylamine having a linear range of 15–1500 μg/L with LOD 0.12 μg/mL^14^. In another study conducted by Huijuanyu et al., an IDA based method for the fluorescence “switch-on” assay was established for the detection using GC5A. F1 reporter pair with LOD and a range of 8.98 mM and 0–1.22 mM, respectively^15^. All these methods described here are expensive and time-consuming; therefore, a need has been felt to develop rapid and point-of-care (POC) methods for fast detection of TMAO. Sensors are an alternative approach for timely detection of TMA or TMAO as they possess the merit of being quick, affordable, sensitive, specific, user friendly, robust, hassle-free, deliverable and able to be fabricated, miniaturized and used as POC devices. MIP based sensors offer all these properties that’s why there is a need to be researched in this area for detection of TMAO. Therefore, the present work described here can easily overcome these limitations and exhibit good results and superiority in terms of sensitivity, linear range, and LOD, as compared to works, reported previously on the detection of TMAO, shown in Table 1.

**Table 1:** Comparison of biosensing platform of MIP/ITO electrode with previously reported techniques towards TMAO detection.

Method	Technique	Electrode	Linear range	LOD	Sensitivity	Response time	References
GC–MS	SPME	**–**	14.9–956 μ mol/l	–	14.9 μ mol/l	–	^9^
Liquid chromatography	SPE	–	5.0–50.0 μg/mL	0.05 μg	–	–	^10^
Chromatography	UPLC-M/MS	–	15–1500 μg/L	0.12 μg/L	–	6 min	^14^
Chromatography	LC-SIMs		15–944 pg/μL	115 pg/mL		5 min	^13^
Fluorescence	IDA	GC5A	0–1.22 mM	8.98 μM	–	–	^15^
Chromatography	FIGD-IC	–	40–600 nmoldm^−3^	1.35 nmoldm^−3^	–	–	^59^
Electrophoretic		Indirect UV detection	0.025–2.5 mM	2.5 mM	–	–	^60^
Chromatography	Ion chromatography	–	1.0–20.0 mg/mL	0.10 mg/L	–	16 min	^61^
Electrochemical	CV	TorA-FDH/GCE	2–110 mM	2.96 nM	14.16 nA/mM	16 s	^12^
Electrochemical	DPV	MIP/ITO	1–15 ppm	1.5 ppm	2.47 µA mL ppm^−1^ cm^−1^	20 min	Present work

FDH: format dehydrogenase; FIGD-IC: flow injection-ion chromatographic technique; GC–MS: gas chromatography-mass spectrometry; GC5A: guanidinium-modified calix [5] arene; GCE: glassy carbon electrode; IDA: indicator displacement assay; LC-SIMs: liquid chromatography-selective ion monitoring; SPE: solid phase extraction; SPME: solid phase microextraction; TorA: TMAO reductase; and UPLC-M/MS.

Several research reports have been published on molecularly imprinted polymers (MIPs)^16–21^, which can be used as artificial receptors for making electrochemical sensors to detect small molecules, helping in diagnosing the disease with excellent sensitivity and performance. In biosensors, the biological receptors have limitations to detect analyte by environmental parameters. Molecular imprinting based on the electrochemical approach has been shown to be an appealing strategy for development of advanced sensors as these are employed as bio-mimicking measurement system and are easy to construct as well as they have a low cost. MIPs/surface imprinted polymers (SIPs) have been proved remarkable in creating artificial receptors. They possess unique physical and chemical stability in making specific cavities for binding analytes such as TMAO in the polymeric matrix. In comparison to synthetic receptors, as in the case of MIP, biological receptors were also used widely in chemo/biosensors, but they involve complex protocols, high cost, and poor stability^22–24^. Because of these limitations, the recent trend can be seen in MIP’s preference for making artificial recognition receptors in sensor development^25^. MIPs offer rapid, inexpensive, and selective receptors to make electrochemical/optical sensors that seem suitable to promptly detect small metabolites like TMAO. Among the various conducting polymers, Polypyrrole (PPy) is one of the most studied materials and frequently used conducting polymer in developing MIP based electrochemical sensors/biosensors due to its facile synthesis, high electrical conductivity, suitable redox properties, good biocompatibility, environmental stability, electrochemical properties^26–30^ and easier polymerization procedure compared to other conducting polymers. In addition to its rapid electrochemical response, low cost, wide dynamic range, low detection limits; PPy is advantageous due to its capability of imprinting biomolecules at room temperature without denaturation and conformational change. Both low molecular weight molecules and high molecular weight molecules can be determined via MIP modified PPy polymer. Also, PPy can be exploited in order to increase the sensitivity of electrochemical detection based analytical systems and electrochemically and chemically stable conjugated chains are formed during the polymerization of PPy. The PPy-based MIP for the electrochemical detection of the ophylline (a drug used in the therapy of respiratory diseases)^31^, tryptophan enantiomers^32^, bovine leukemia virus^33^, ascorbic acid and for other analytes has already been reported^34, 35^. Also, there is significant work published (Table 2) for the fabrication of various electrochemical sensors/biosensors using pyrrole as a monomer due to the aforementioned unique properties towards different analytes’detection^36–49^. However, there is no single report exists on the PPy-based MIP system for detecting any gut metabolite, including TMAO. Thus, there is a broad scope to develop a MIP based sensing platform for the detection of gut microbiota-derived metabolites, including TMAO.

**Table 2:** Various pyrrole based MIP electrochemical sensors developed for different analytes.

Analyte/template	Functional monomer	Crosslinker/polymerization method	Initiator	Solvent	Detection method/Range/LOD	Reference
p-nonylphenol	Pyrrole + TiO_2_	No crosslinker/chemical	FeCl_3_	Isopropanol	Electrochemical/range-1.0*10^−8^ to 8*10^−5^ mol/L/LOD: 3.91*10^−9^ mol/L	^40^
4-Ethylphenol	Pyrrole	No crosslinker/ Electropolymerization	LiClO_4_	BR Buffer	Electrochemical DPV/Range-0.2 to 34.8 μM/LOD- 0.1 μM	^41^
Myo-inositol	Pyrrole	No crosslinker/electropolymerization	LiClO_4_	KCl	Electrochemical/ Range-1.0 × 10^−1^0 mol L^−1^ to 1.0 × 10^−8^/LOD-7.6 × 10^−11^ mol L^−1^	^42^
Fluoxetine	Pyrrole	No crosslinker/ Precipitation polymerization	Copper (II) chloride	Methanol	Solid phase extraction/Range-10^−7^–10^−8^ M/LOD-6.56 × 10^−9^ M	^43^
1,4-dihydroxyanthraquinone	Pyrrole	No crosslinker/ Precipitation polymerization		BR buffer + acetonitrile	Electrochemical/Range-10 nmol L^−1^ to 100 mmol L^−1^/LOD-4.15 nmol L^−1^	^44^
Phenothiazine	Pyrrole	Electropolymerization/no crosslinker	NaClO_4_	Acetonitrile–water	Electrochemical/Range-1–300 mmol L^−1^ and 0.5–10 mmol L-1/LOD-3 10^−7^ mol L^−1^	^45^
Sulfadimethoxine	Pyrrole	No crosslinker/ Precipitation polymerization	LiClO_4_/TBAP	BR Buffer	Electrochemical/Range-0.15 to 3.7 mM/LOD-70 μM	^46^
Caffeine	PTEOS, Pyrrole	TEOS	HAuCl_4_	PBS + KCl	Electrochemical/Range-2.0 to 50.0 and 50.0 to 1000.0 nmol L^−1^/LOD-0.9 nmol L^−1^	^47^
Trimethoprim	Pyrrole	No crosslinker/ Precipitation polymerization	LiClO_4_	Water	Electrochemical/Range-1.0 × 10^−6^ to 1.0 × 10^−4^/LOD-1.3 × 10^−7^ M	^48^
Dopamine	Pyrrrole	No crosslinker/ Precipitation polymerization	LiClO_4_	MeOH/AcCOOH Extraction solution	Surface acoustic wave sensor/Sensitivity- ≈550 Hz/mM/LOD- ≈ 10 nM	^49^
CA-125	Pyrrole	No crosslinker/ Precipitation polymerization		KCl	Electrochemical and SPE sensor/Range- 0.01 and 500 UmL^−1^/LOD- 0.01 UmL^−1^	^50^
Quercetin	Pyrrole + b-CD/AuNPs/GR	No crosslinker/ Precipitation polymerization		KCl	Electrochemical/Range- 1.0 × 10^−9^ to 1.0 × 10^−6^ mol L^−1^/LOD-1.0 × 10^−1^0 mol L^−1^	^51^
Triacetonetriperoxide	Pyrrole	No crosslinker/ Precipitation polymerization	LiClO_4_	Acetonitrile	Electrochemical/Range- 82–44,300 µg·L^−1^/LOD- 26.9 μg·L^−1^	^52^
Isoproturon	Pyrrole	No crosslinker/precipitation polymerization	LiClO_4_	Ethanol + water	Electrochemical/LOD- 0.5 μg L^−1^ in mili Q water, 2.2 μg L^−1^ in real samples	^53^

Our aim of the present work is to develop a sensitive, cheap, and reproducible MIP based method that would allow quantification of TMAO at lower quantities (1–15 ppm). Here, we report the first and innovative electrochemical TMAO sensor based on the MIP. The MIP was developed based on the polymer template, PPy, which has a recognition site for TMAO, and electrochemical differential pulse voltammetry (DPV) detection is used to measure the TMAO. This method of TMAO detection has the following steps: (a) synthesis of polymer template, PPy by chemical oxidation, with and without the presence of TMAO; (b) filtration and drying of PPy-TMAO and PPy powders and; (c) electrochemical DPV detection of TMAO at different concentrations ranging between 1 and 15 ppm. The experimental conditions were optimized and all the electrochemical studies for the detection of TMAO were done using cyclic voltammetry (CV) and differential pulse voltammetry (DPV) in PBS containing [Fe(CN)_6_]^3−/4−^ as an active redox species. This fabricated MIP/ITO electrode exhibited a comprehensive linear response in the concentration range of 1–15 ppm, high sensitivity of 2.47 μA mL ppm^−1^ cm^−2^, a lower detection limit of 1.0 ppm with a fast response time of 20 min when compared to other reported sensors and conventional techniques for TMAO detection. The developed sensor was used for TMAO measurement in the body fluids such as urine, and recovery of TMAO was observed from 100.8 to 105.7% with an RSD of 0.6–3.9%. Finally, the interferents study was also performed to ensure its selectivity.

## Results and discussion

### Fourier transform infrared spectroscopy analysis

Figure S1 (Supplementary data) shows the FTIR spectra of NIP (a), MIP (b), PPy-TMAO (c), and TMAO (d). The typical peaks obtained in (a), (b), and (c) are tabulated in Table 3 with their respective bond assignments. All peaks, as mentioned in Table 3, have appeared in all three spectra (a), (b), and (c) with slight differences as the matrix corresponds to PPy. The peaks visible in the spectrum (d) of TMAO (chemical formula C_3_H_9_NO) are as follows: the broadband around 3500 cm^−1^ was assigned to N–H stretch in diluted solution; a small peak at 2930 cm^−1^ was due to CH anti-symmetric stretch, and the weak peak at 2850 cm^−1^ corresponds to CH_3_–O or N stretching modes. The band at 1690 cm^−1^ was assigned to C = N stretch. The small peaks at 1424 cm^−1^ and 1377 cm^−1^ were due to –OH bending, aliphatic -CH_3_ symmetric deformation, respectively. The N–O band of N-oxides appeared at 1238 cm^−1^^50^. Besides this, few more weak peaks appeared at 780 cm^−1^, 640 cm^−1^, and 570 cm^−1^ were of –CH bending or out of plane vibrations; finally, the broadband between 400 and 560 cm^−1^ corresponds to C–N–C in amines. When TMAO was present with PPy in the spectrum (c), a weak peak of C–N–C appears along with the formation of fewer peaks between 450 and 560 cm^−1^, which disappeared in the spectrum (a). The vast stretch at 1650–1700 cm^−1^ of C=N stretch becomes sharp in (b) and (c) showing the deformation after incorporation of TMAO. The integration of TMAO into the PPy matrix was observed from the corresponding peaks at 2930 cm^−1^, and 2850 cm^−1^ in the spectrum (c). The removal of the template was confirmed by the disappearance of these peaks in the spectrum (b). From these results, it can be concluded that the template TMAO was successfully incorporated into the PPy matrix and was removed in MIP. However, the incorporation of TMAO did not change the PPy structure showing that the template was not attached to the polymer matrix by forming strong chemical bonds. It was attached through weak hydrogen bonds*.* The significant changes in IR spectra are shown in the rectangle box in Figure S1.

**Table 3:** FTIR transmission peaks and their assignment in PPy which confirms the formation of MIP, NIP and PPy-TMAO through different bonds.

S. no	Peak position (cm^−1^)	Corresponding bond
1	674	C = C–H bending
2	786	C–H out of plane bending
3	906	CH_2_ out of plane wagging bond in CH = CH_2_
4	966	C–C out of plane ring deformation
5	1036	C ring breathing vibration in cyclic compounds
6	1298	C–H and N–H in-plane deformation
7	1169 and 1462	C–N stretching vibration
8	1540	C = C stretching vibration in pyrrole ring
9	1650–1700	C = N stretch

### SEM analysis

The scanning electron microscopy (SEM) imaging was employed to study the surface morphologies of NIP, MIP, and MIP-TMAO. Figure S2 shows the SEM images of (a) NIP, (b) PPy-TMAO, and (c) MIP. It is clear from the pictures that the NIP and PPy showed a combined fibrous kind of structure. This is because the method followed for the synthesis of PPy was the interfacial polymerization method, which leads to the formation of fibers. The fibers were not visible individually, but they were combined. When the template (TMAO) was incorporated into the PPy matrix during polymerization, the polymer's morphology showed a lot of difference with higher roughness with lumps present on the surface, as shown in the image (b). After the template was removed, the morphology was changed, as shown in the image (c). As TMAO is a tiny molecule-sized about 1.5 nm, it cannot be seen in SEM. However, the morphological changes were observed in Figure S2, which can be correlated with FTIR results confirming that the morphological changes were due to the template removal after ultra-sonication. Further, the detection of TMAO was carried out using electrochemical DPV studies.

### Electrochemical studies

#### pH study

**Figure 1:**
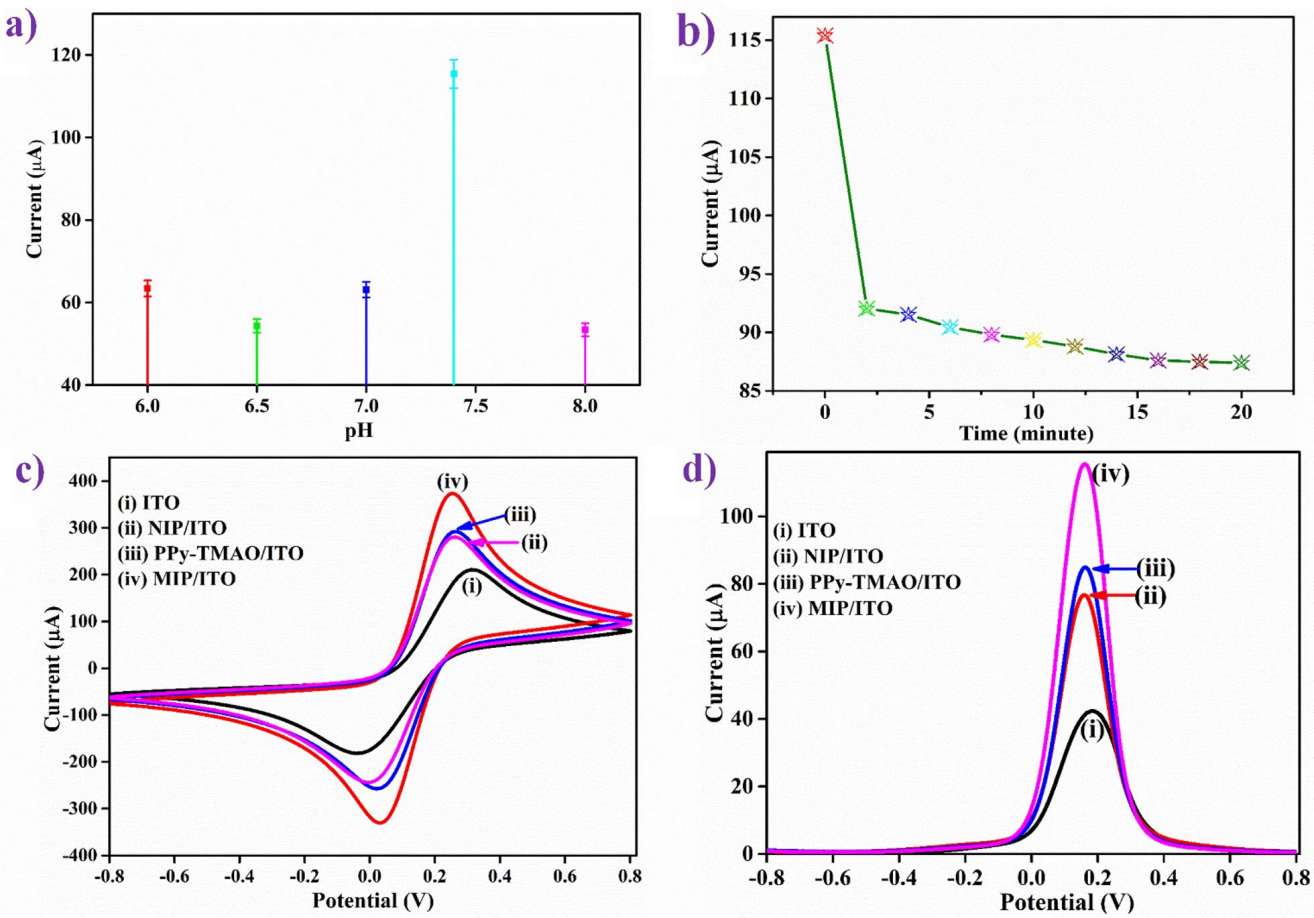
Electrochemical studies of MIP/ITO electrode (**a**) effect of pH; and (**b**) response time. Comparative (**c**) CV and (**d**) DPV study of ITO (curve i); NIP/ITO (curve ii); PPy-TMAO/ITO (curve iii); and TMAO/ITO (curve iv) in PBS (0.2 M, 0.9% NaCl) containing 5 mM of [Fe(CN)_6_]^3−/4−^.

The electrochemical reaction between an analyte and MIP is usually affected by pH of the buffer. Hence, buffers (PBS containing [Fe(CN)_6_]^3−/4−^) of pH ranging from 6.0 to 8.0 were used to analyze the impact of pH on MIP/ITO electrode, in the potential range of -0.8–0.8 V. Figure 1(a) shows that as the pH value increased from 6.0 to 7.4, the DPV peak current increased and then decreased at the pH 8.0. This observation can be attributed to the fact that at pH 7.4, the MIP-based electrochemical sensor's imprinted cavities combine with more template (TMAO) molecules^51^. Thus, the MIP/ITO electrode was found to exhibit the maximum peak current at pH 7.4 (physiological pH). Therefore, pH 7.4 of PBS containing [Fe(CN)_6_]^3−/4−^ was used as the medium for further electrochemical response studies.

#### Response time study

When the analyte, TMAO interacts with the MIP/ITO electrode surface, the attachment depends on the incubation time. Therefore, for the investigation of the incubation time required for the interaction between TMAO (at 12 ppm) and MIP/ITO electrode, response time studies were conducted in pH 7.4 PBS containing [Fe(CN)_6_]^3−/4−^ in the range from 0 to 30 min at the interval of 2 min (Fig. 1b) using DPV. It was observed that DPV peak current is inversely proportional to the incubation time from 0 to 19 min, but after that, it showed no significant change. These observations led to the conclusion that the interaction of TMAO withMIP/ITO electrode took approximately 20 min. Thus, an optimum time of 20 min was given to the interaction of TMAO and MIP/ITO electrode before taking every reading for the electrochemical response studies.

#### Electrode study

The fabricated MIP/ITO electrode was used to detect TMAO. The electro-activity of the electrodes MIP/ITO, NIP/ITO, and PPy-TMAO/ITO was studied through CV and DPV studies. Figure 1(c) shows CV response of (i) ITO (ii) NIP/ITO (iii) PPy-TMAO/ITO and (iv) MIP/ITO electrodes at a scan rate of 50 mV/s in the potential range of -0.8 to 0.8 V in PBS containing [Fe(CN)_6_]^3−/4−^ as a redox species. It was observed that in comparison to ITO (210.44 μA), the NIP/ITO, MIP/ITO and PPy-TMAO/ITO electrodes manifested more current values, (i.e., these were more conductive) and this can be assigned to PPy being a conducting polymer, which is highly electroactive that aids in the conduction of ions. As shown in Fig. 1(c), the peak current in the CV curve NIP/ITO and incorporation of TMAO into the PPy matrix (i.e., PPy-TMAO) showed almost the same values with a slight shift in the peak voltages. NIP/ITO electrode showed the lowest peak current (280.42 μA; curve ii). The incorporation of TMAO into PPy, i.e., PPy-TMAO/ITO electrode, increased this current slightly (291.99 μA; curve iii).

Further, the increase in peak current was observed after the removal of TMAO from PPy-TMAO by ultra-sonication to form MIP/ITO electrode (373.56 μA; curve iv), since TMAO acts as an oxidant. Moreover, when the template was removed, there were more free-NH groups available in PPy that leads to an increase in the electro-activity and thereby resulting in an escalation in the CV peak current of MIP. The change in the peak currents in the electrode study, and the changes in the FTIR spectra indicated that TMAO was successfully removed from the MIP. The DPV scans were also coherent with the trend of current values observed in CV scans as shown in Fig. 1(d).

#### Scan rate study

**Figure 2:**
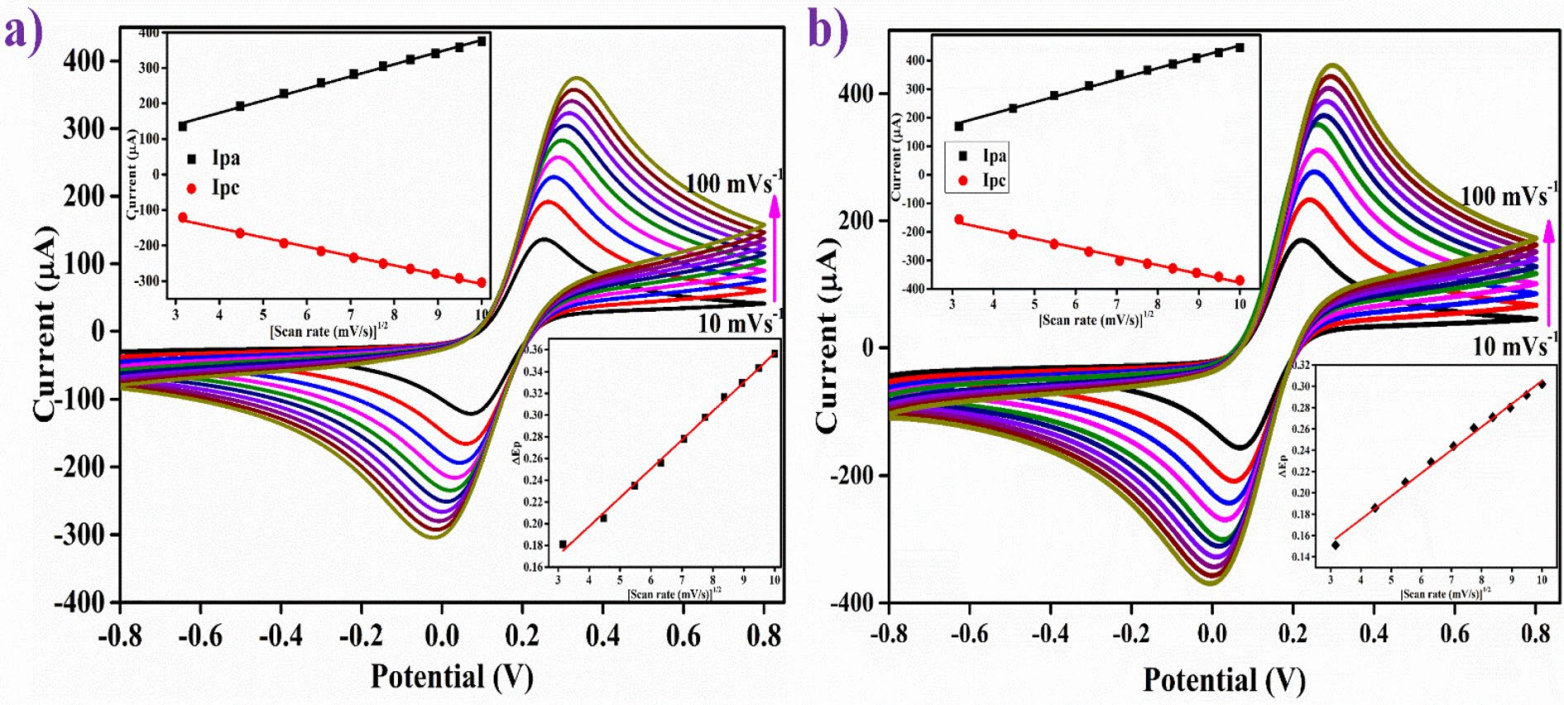
CV plots at different scan rates (10–100 mV/s) of (**a**) NIP/ITO and (**b**) MIP/ITO electrodes in pH 7.4 PBS containing 5 mM of [Fe(CN)_6_]^3−/4−^. Corresponding insets showed peak current (I_pa_ and I_pc_) vs. √*v* (upper) and peak potentials (E_pa_ and E_pc_) vs. √*v* (lower) for respective electrodes.

Figure 2(a,b) shows the CV scans that were run to examine the electro-kinetics of NIP/ITO and MIP/ITO electrode occurring at the interface between the surface of the electrode and electrolytes in the solution using a scan rate of 10 to 100 mV/s. The I_pa_/I_pc_ ratio for the MIP/ITO electrode was determined to be 0.92. The ratio is close to 1, which suggests the quasi-reversible electron transfer kinetics^52,53^. The I_pa_/I_pc_ ratio for the NIP/ITO electrode was determined to be 0.82, which pointed towards the progression of an irreversible electron transfer between the electrode and the medium [as shown in the upper inset of Fig. 2(a,b)]. The slopes and intercepts for these I_pa_ and I_pc_ curves can be determined from equations (S1) to (S4).

Moreover, the difference between the cathodic peak (E_pc_) and the anodic peak potential (E_pa_), represented by ΔE_p_ [ΔE_p_ = E_pa_ − E_pc_], was found to be 0.278 V for the NIP/ITO electrode and 0.233 V for the MIP/ITO electrode. Furthermore, the difference between the peak potentials E_pc_ (cathodic) and E_pa_ (anodic), ΔE_p_ exhibits a linear relationship with the progression in the scan rate (ʋ) according to the equations (S5) and (S6). This is confirmed in Fig. 2(a,b) (lower inset), which shows the plot between ΔE_p_ and the square root of the scan rate (√ʋ). The existence of a linear relationship between ΔE_p_ and √ʋ indicates an easy and uncomplicated electron transfer between the electrodes and the medium.

The peak currents I_pa_ (anodic) and I_pc_ (cathodic) were also found to increase linearly with the square root of the scan rate (√ʋ) for both the electrodes, thus indicating the dependency of the electrochemical reaction on the diffusion of the electroactive species occurring at the electrode/electrolyte interface. In other words, the electrochemical reaction was a diffusion-controlled process^52,54^. The scan rate studies of both MIP/ITO and NIP/ITO electrodes showed that the peak of oxidation current shifted towards the positive potential. Similarly, the peak of reduction current also shifted towards the negative potential. Also, the rise in peak potential (E_pa_ and E_pc_) with an increase in the scan rate recommends a slow transfer of electrons at the interface [Fig. 2(a,b) (lower inset)]^55^.

The movement of electrons was examined and analyzed by the measurement of kinetic interface factors such as the surface concentration of the redox probe of the electrode (I^∗^), diffusion constant (D), surface area (A_e_) and electron transfer rate constant (K_s_) for both NIP/ITO as well as MIP/ITO electrode. The interface kinetic parameters corresponding to electrode are set out in Table 4. The diffusion coefficient (D) at the bioelectrode surface and electrolyte interface having redox species [Fe(CN)_6_]^3−/4−^ was determined by employing the Randles–Sevcik equation (S7)^54^; where I_p_ (I_pa_ or I_pc_) symbolizes the peak current of electrodes, ‘n,’ is the number of electrons involved in the redox event (= 1 here), A is the surface area of bioelectrode (0.25 cm^2^), D is the coefficient of diffusion (cm^2^ s^−1^), C is the concentration of electrolytes, and v is scan rate (Vs^−1^).

**Table 4:** Electrochemical parameters of NIP/ITO and MIP/ITO electrodes. The movement of electrons is very crucial in electrochemistry which was examined and analyzed by the measurement of kinetic interface factors such as the surface concentration of the redox probe of the electrode (I^∗^), diffusion constant (D), surface area (A_e_) and electron transfer rate constant (K_s_) for both NIP/ITO as well as MIP/ITO electrode.

Electrode	m (V)	A (cm^2^)	Ks (s^−1^)	γ* (mol/cm^2^)	D (cm^2^ s^−1^)
NIP/ITO	0.2784	0.25	0.538	2.43 × 10^−8^	1.15 × 10^−12^
MIP/ITO	0.2295	0.25	0.444	3.02 × 10^−8^	1.77 × 10^−12^

The higher value of D demonstrated for MIP/ITO electrode (1.77 × 10^−12^ cm^2^ s^−1^) indicates a higher rate of transfer of electron at the electrolyte /electrode interface in comparison to NIP/ITO (1.15 × 10^−12^ cm^2^ s^−1^).

The unmodified electrodes, i.e., NIP/ITO and MIP/ITO bioelectrodes’ efficient and effective electroactive surface area (A_e_) was determined by putting the value of D calculated using the equation of Randles-Sevcik^54^ (S8); where *S,* the slope of the linear curve derived from the plot between I_pa_ and the square root of scan rate (v^1/2^) [in (mV/s)^1/2^]. The calculated values are specified in Table 4. As is clear from Table 4, the value of MIP/ITO bioelectrode’s electroactive area (A_e_) is 0.25 mm^2^, which revealed that these are more suitable owing to more reaction sites/unit volume due to the formation of cavities as compared to NIP/ITO electrode (0.25 mm^2^)^56^.

The linear relationship between the I_pa_value of the scan rate (R^2^ = 0.995) for NIP/ITO and MIP/ITO electrode showed the steady phase of electroactive species of electrodes. Furthermore, the surface concentration of the absorbed electroactive ionic species (I* in mol cm^2^) for the corresponding electrodes has been calculated using Brown-Anson equation^53^ (S9); where F- the Faraday constant (96,485 C mol^−1^), T, room temperature (300 K), and R, gas constant (8.314 mol^−1^ K^−1^). The approximate value of surface concentration (I*) for the MIP/ITO electrode (3.02 × 10^−8^ mol cm^2^) is higher as compared to the NIP/ITO electrode (2.43 × 10^−8^ mol cm^2^), which reveals an enhancement in the electro-catalytic behavior at its surface due to the presence of cavities.

The electron transfer reversibility kinetics depend on the scan rate as well as on heterogeneous electron transfer rate constant (K_s_). The values of K_s_ for NIP/ITO and MIP/ITO electrode are 0.44 s^−1^ and 0.53 s^−1^, respectively, estimated by Laviron Equation (S10); where, m- the peak-to-peak separation of potentials (V). The MIP/ITO electrode had higher K_s_ value (given in Table 4), indicating that the electron exchange is fast between the surface of the electrode and electrolyte redox species^57^.

Table 4 provides the calculated values of various electrochemical parameters of the ionic species of both the electrodes: Peak currents—cathodic (I_pc_) and anodic (I_pa_), electron transfer rate constant (K_s_), coefficient of diffusion (D), the average surface concentration of the absorbed electroactive species (I*) and electroactive surface area of the electrode (A_e_). The interface kinetic parameters for the MIP/ITO electrode have a higher value than the NIP/ITO electrode, which suggests that the cavities generated after removal of analyte TMAO significantly contribute to the enhancement of electron transfer at the bioelectrode and electrolyte interface.

#### Electrochemical response studies

**Figure 3:**
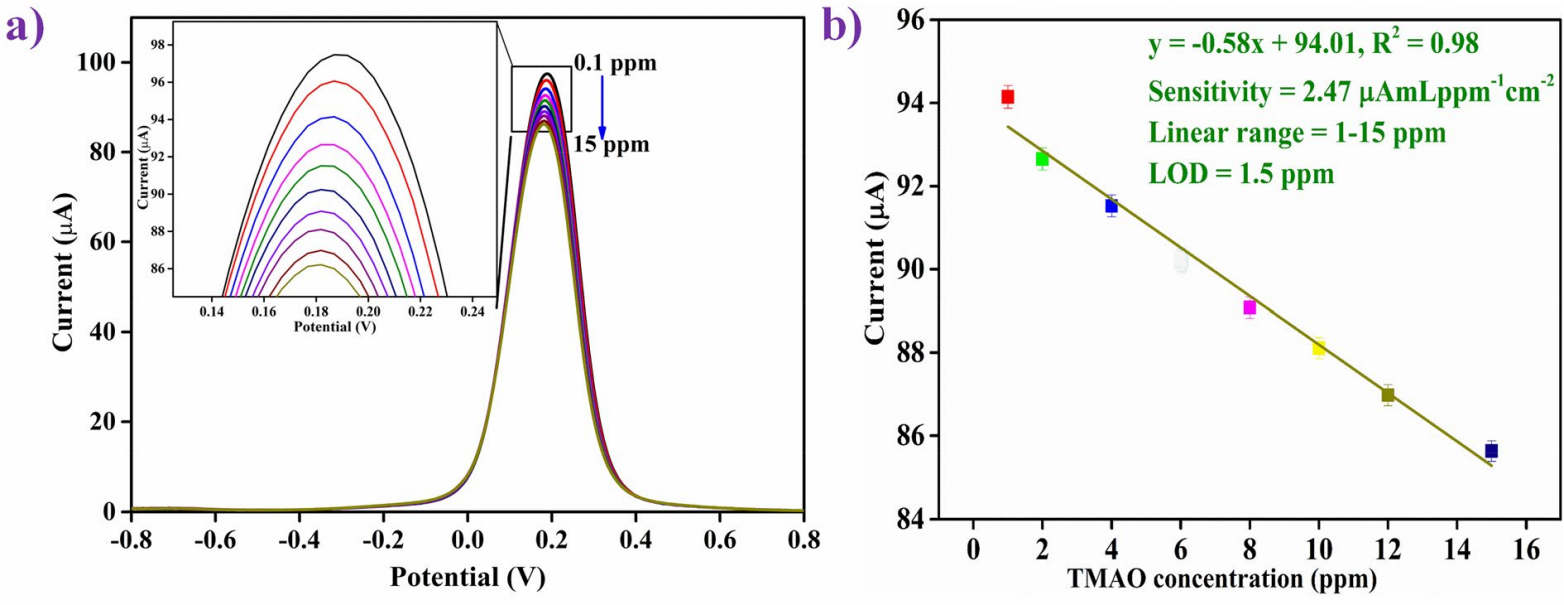
(**a**) DPV response studies of MIP/ITO electrode as a function of TMAO concentration (0.1–15 ppm mL^−1^) in pH 7.4 of PBS (0.2 M, 0.9% NaCl) containing [Fe(CN)_6_]^3-/4-^; (inset) magnified view of peak currents (**b**) calibration curve between the magnitude of peakcurrent and concentration of TMAO (ppm mL^−1^).

DPV is an extensively used and suitable technique for studying the electrochemical response due to higher current sensitivity than linear sweep voltammetry and CV and because of potential pulse programming^58^. Hence, the response study of MIP/ITO electrode was conducted using the DPV technique in the potential range from −0.8 V to + 0.8 V for the detection of analyte TMAO by successive incorporation of various concentrations of TMAO from 0.1 to 15 ppm (0.1, 0.5, 1, 2, 4, 6, 8,10, 12 and 15 ppm) in PBS containing [Fe(CN)_6_]^3−/4−^ as the redox species with an incubation time of about 20 min at room temperature. It was found that there is a linear decrease in peak current, which is proportional to the addition of each TMAO concentration from 1 to 15 ppm, and after adding 15 ppm, the current became constant (Fig. 3a). The TMAO cavities present in the MIP deposited on the ITO electrode (i.e., on the MIP/ITO electrode) were saturated with a TMAO concentration of 15 ppm. Thus, no further decrease in current was observed above this saturating concentration (i.e., 15 ppm). The lowest TMAO concentration capable of detection was 1.0 ppm. Therefore, 1.0 ppm is the lower limit of detection of the developed sensor. The enlarged peak positions were shown in the inset of Fig. 3(a).

Figure 3(b) represents the calibration curve, which shows the relationship between the change in peak current and different TMAO concentrations performed by the DPV. The result of the calibration curve showed clearly a linear and an inverse relationship between the response peak current of the MIP/ITO electrode and the concentration of TMAO in a wider linear range of 1-15 ppm. Equation S11 shows the linearity curve parameters. The experiments were repeated four times, and the error bars show the standard deviation.

The sensitivity of the fabricated MIP/ITO electrode was determined by the ratio of the slope of the curve to the surface area (0.25 cm^2^) of the electrode^53^, and was found to be 2.47 μA mL ppm^−1^ cm^−2^ with R^2^ of 0.981 of the linear plots as shown in Fig. 3(b). The limit of detection (LOD) for the MIP/ITO electrode was obtained to be 1.0 ppm mL^−1^, which is one of the lowest achieved until now when compared to the similar platform [2.96 nM]^12^ and other conventional techniques [0.121 μg/L^14^; 115 pg/mL^13^; 8.98 μM^15^; 1.35 nmol dm^−3^^59^; 2.5 mM^60^; and 0.10 mg/L^61^] for TMAO detection. The LOD has been calculated using the formula given in Eq. (S12)^53^; where *σ* represents the standard deviation of the blank electrode, and ‘k’ is the slope of the linear calibration curve given in Fig. 3(b). These results reveal the higher sensitivity (2.47 μA mL ppm^−1^ cm^−2^), low LOD (1.0 ppm mL^−1^), and wide linear detection range of 1–15 ppm with a fast response time of ~ 20 min for the TMAO detection and found superior among the previously reported works. All the measurements and experiments were performed in quadruplicates to confirm the repeatability and reproducibility using the developed sensor. Table 1 gives a comparative study of some previously reported work based on sensors and other techniques towards TMAO detection^13–15, 59–62^.

**Figure 4:**
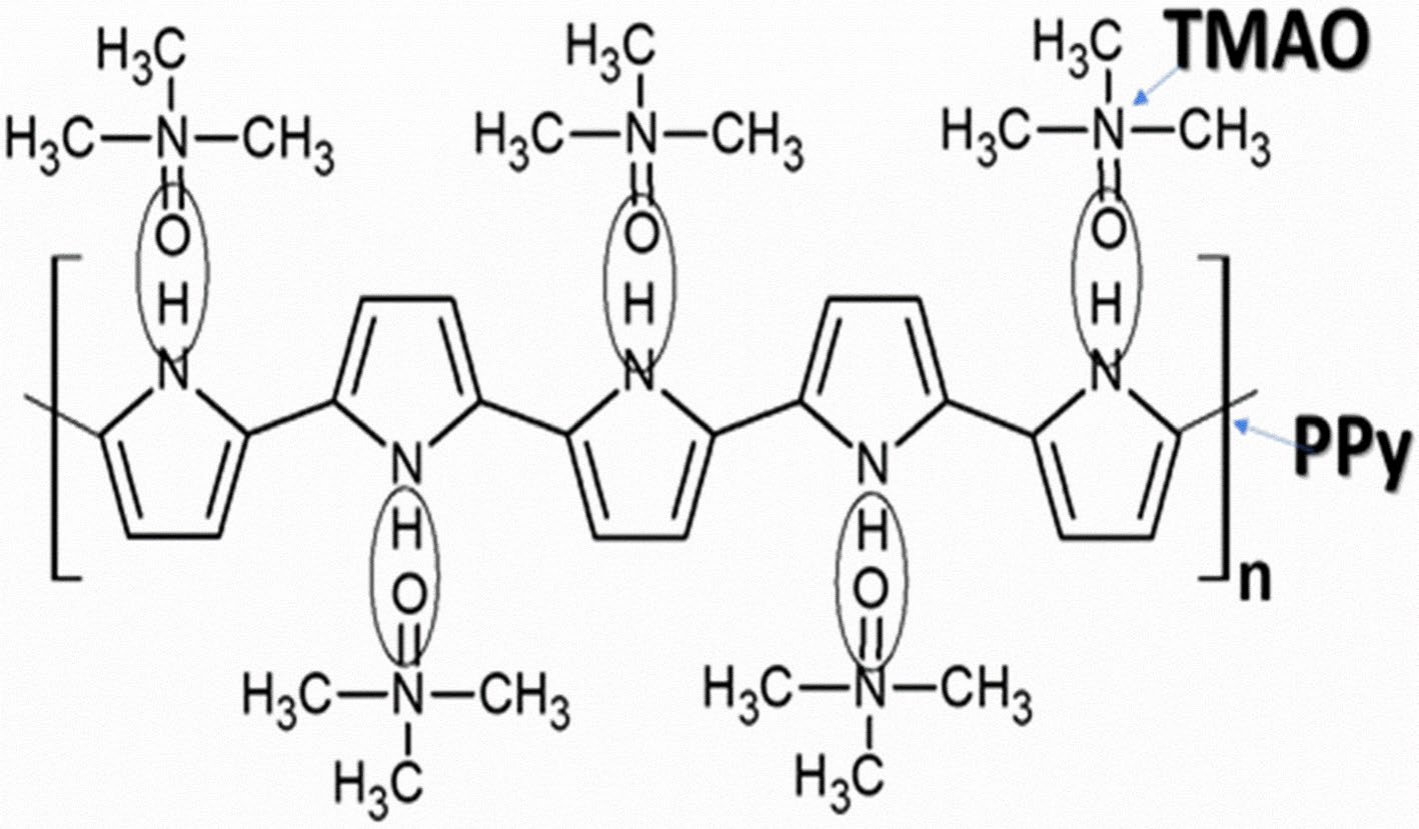
Chemical interaction between TMAO and PPy. The interaction between the N-oxide of TMAO and PPy occurs by formation of hydrogen bonds between NH of PPy and oxygen atoms in N-oxides. When TMAO was added at different concentrations, the NH groups started binding with the oxygen of TMAO, thereby leading to a decrease in the electro-activity and peak current in the response study. The image was created using ACD Labs Freeware, version 2017 (https://www.acdlabs.com/index.php).

In the above studies, it was found that there is no such increase in the DPV peak current with the incorporation of TMAO into the PPy matrix. It was found that the removal of TMAO from the PPy matrix to form MIP showed an increase in the peak current values. When TMAO was introduced into the MIP matrix again, the peak current was found to decrease. Therefore, the sensing of TMAO shows a decrease in the DPV peak current. TMAO directly acts as an oxidant. The interaction between the N-oxide and PPy occurs by formation of convergent hydrogen bonds between NH of PPy and oxygen atoms in N-oxides. This implies that the template TMAO was attached to the PPy matrix through hydrogen bonding, which helped in the simple removal of the template through ultra-sonication (Figure S1). Further, when TMAO was added at different concentrations, the NH groups started binding with the oxygen of TMAO, thereby leading to a decrease in the electro-activity and peak current in the response study. The sensing mechanism of the developed MIP is shown in the Fig. 4.

#### Control study

**Figure 5:**
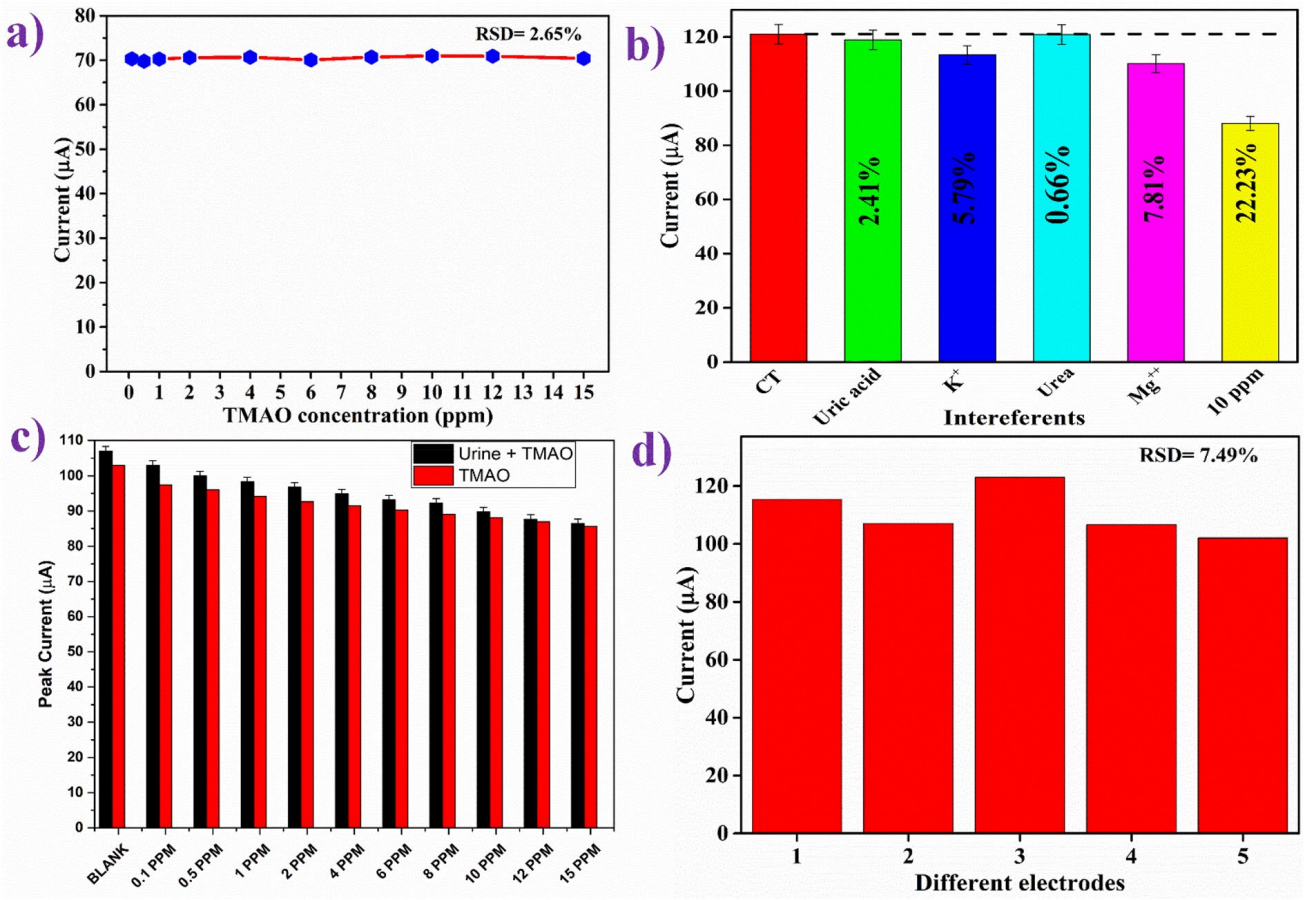
(**a**) Control experiment using DPV of NIP/ITO electrode as a function of TMAO concentration (0.1–15 ppm mL^−1^); (**b**) Effect of potential interferents on the response study of MIP/ITO electrode; (**c**) Spiked-in sample response of MIP/ITO electrode in comparison with response study of TMAO; and (**d**) Reproducibility study of MIP/ITOelectrode fabricated under similar condition.

NIP/ITO electrode, having no cavities, should not interact with the antigen, i.e., TMAO. A control experiment was conducted to check and confirm the absence of cross-reactivity of the NIP/ITO electrode with TMAO and also to determine the electrochemical response of the NIP/ITO electrode as a function of different TMAO concentrations (Fig. 5a). There were no considerable changes in the DPV current response of the NIP/ITO electrode with the increasing concentrations of TMAO. This proved the specific interactions of the TMAO with the cavities present on the MIP/ITO electrode surface and that it has almost no interaction with the NIP/ITO electrode. Therefore, there are no substantial changes in the electrochemical current response.

#### Interferents study

Specificity is an essential and vital parameter for most sensors as the non-specific bindings can hinder and mislead the detection result. Thus, interferent studies of the fabricated MIP/ITO electrode were performed using DPV in PBS containing [Fe(CN)_6_]^3−/4−^ by prior-incubation with various impurities present in human urinein particular concentration such as Uric acid [0.03 g 100 mL^−1^], urea [2 g 100 mL^−1^], potassium ions (K^+^) [0.15 g 100 mL^−1^], and magnesium ions (Mg^2+^) [0.015 g 100 mL^−1^]. The observed currentresponse of the MIP/ITO electrode with differentinterferents is shown in Fig. 5(b). It was noticed that no significant change in peak current magnitude happened due to the introduction of the various interferents. Although, thepeak current decreased to 99.09 μA upon the addition of TMAO (15 ppm mL^−1^), indicating that the MIP/ITO electrode specifically interacted with TMAO and the potential interferents in urine had a negligible effect on the response.

#### Spiked sample and reproducibility study

A prepared spiked urine sample served as the real sample and was tested to validate the practicability of fabricated MIP/ITO electrode using the DPV technique in PBS containing [Fe(CN)_6_]^3−/4−^. For this study, 10 μL of each sample was added to 4 ml of PBS, and the obtained results are shown in Fig. 5(c). From the Fig. 5(c), it can be seen clearly that the DPV peak currents decreased sequentially with the sequential increase in the concentration of TMAO in the spiked sample. To determine the percent recovery and standard deviation (RSD), measured current responses of the samples were investigated with the known concentration of spiked TMAO (0.1–15 ppm) sample solutions. The % of TMAO recovery and RSD acquired from spiked urine samples was 100.8–105.7% and 0.6–3.9%, respectively, as given in Table 5. The TMAO recovery value and the RSD value are reasonably good, establishing the accuracy and reliability of the sensor accuracy for application in real-time diagnosis of samples. Since gut metabolites appear in urine and serum samples, detection in any of these by this sensor will be helpful.

**Table 5:** Relative standard deviation (RSD) and recovery percentage of TMAO from human urine samples using MIP/ITO electrode.

TMAO added to the urine sample (ppm)	DPV peak current for TMAO (μA)	DPV peak current of spike-sample (μA)	Relative standard deviation (RSD %)	Recovery (%)
0	103	107	2.69	103.88
0.1	97.473	103	3.90	105.67
0.5	96.069	100	2.84	104.09
1	94.147	98.3	3.05	104.41
2	92.651	96.8	3.10	104.47
4	91.522	94.9	2.56	103.69
6	90.24	93.2	2.28	103.28
8	89.08	92.3	2.51	103.61
10	88.104	89.8	1.35	101.92
12	86.975	87.7	0.59	100.83
15	85.63	86.5	0.71	101.01

Five separate electrodes with a consistent total area were manufactured under identical conditions, and their electrochemical behaviors were reported using DPV to assure reproducibility. It was found that all five electrodes exhibited comparable current. Thus, the method has good reproducibility. The average/mean value of the current was calculated to be ~ 119 μA. Each measurement was performed in triplicates for each electrode, and after calculating the mean and standard deviation, the error bars were included. The prepared electrodes showed high reproducibility, which is evident by the low value of relative RSD of 7.6%, as shown in Fig. 5(d).

## Conclusions

TMAO has been reported as a possible candidate that could relate microbial dysbiosis to colorectal cancer, Alzheimer’s, cardiovascular, chronic kidney diseases, cerebrovascular, and other diseases. This study presents the successful fabrication of an innovative TMAO-MIP based electrochemical sensor for the first time for the detection of TMAO using the electroactive polymer PPy as the imprinted polymer matrix. TMAO was attached to PPy through hydrogen bonding between -NO and -NH groups, respectively. Since hydrogen bonding is weak, the removal of the template was achieved by ultra-sonication, followed by washing. The template removal was confirmed by FTIR and electrochemical studies, and supported by SEM studies. The electrochemical studies were done using DPV in PBS, containing [Fe(CN)_6_]^3−/4−^ as a redox species. The developed MIP sensor showed a broad linear detection range (1–15 ppm), excellent sensitivity (2.47 μA mL ppm^−1^ cm^−2^), low limit of detection (1.0 ppm mL^−1^), and high selectivity with a fast response time of ~ 20 min for the TMAO detection. This low LOD permitted the sensor to be applied for the direct detection of TMAO in a diluted complex, ‘real’ urine sample. The recoveries with an RSD percentage of the spiked sample were found to be 100.8 – 105.7% and 0.6 – 3.9%, respectively, which reinforces the obtained results. Thus, the MIP based detection will be a promising technique to detect gut metabolites.

## Experimental section

### Material and methods

High purity Trimethylamine N-Oxide (TMAO) was procured from SigmaAldrich. Pyrrole, glucose, uric acid, and urea were purchased from Merck. Ferric chloride (FeCl_3_), hydrochloric acid, ethanol, and sodium chloride (NaCl) were procured from SRL Limited. Sodium hydroxide pellets (NaOH), sodium phosphate monobasic anhydrous (NaH_2_PO_4_), sodium phosphate dibasic dihydrate (Na_2_HPO_4_) potassium ferricyanide (K_3_[Fe(CN)_6_]), potassium ferrocyanide (K_4_[Fe(CN)_6_]3H_2_O) were obtained from fisher scientific. De-ionized water (DI) from the Millipore water purification system was used to prepare the solutions. The ferri-ferro containing phosphate buffer at different pH were made in the lab, as described in the previous report^53,63^. Indium Tin Oxide (ITO) coated glass substrate of 1.1 mm thickness, having a transmittance of 90%, and sheet resistance of 25 Ω sq^−1^ was purchased fromBlazers (U.K). All other chemicals were used without any further purification and were of analytical grade. TMAO solution was prepared at various concentrations from a specific stock solution of TMAO in the range of 0.1 – 15 ppm [0.1 ppm, 0.5 ppm, 1 ppm, 2 ppm, 4 ppm, 6 ppm, 8 ppm, 10 ppm, 12 ppm, and 15 ppm] for the electrochemical response study experiments.

### Synthesis of MIP, NIP, and PPy-TMAO

The interfacial oxidative polymerization was employed for the synthesis of the TMAO imprinted polypyrrole (PPy), in which ferric chloride (FeCl_3_) was used as the oxidizing agent for the oxidative polymerization of pyrrole (C_4_H_4_NCH_3_). Two solutions were prepared in this process – 5 mM pyrrole in 50 mL chloroform (0.1 M) (organic phase) and 5mM FeCl_3_ in 100 mL D.I. containing 1 mL hydrochloric acid (HCl, 1 mM) and 0.1 mL of 1 μM TMAO dissolved in it (aqueous phase). The two solutions – FeCl_3_ solution containing hydrochloric acid and TMAO and pyrrole in chloroform (aqueous and organic phase respectively) were mixed. This was done by carefully transferring the aqueous solution alongside the walls of the beaker containing the pyrrole (in CHCl_3_) solution to let a water-chloroform interface to form. This setup led to the interfacial polymerization of TMAO imprinted PPy. The beaker was left untouched and undisturbed for 12 h continuously at room temperature (25 °C) after coveringwith aluminium foil (to prevent the chloroform from volatilizing). After 12 h, chloroform was carefully removed from the solution. The aqueous component of the solution containing PPy-TMAO was collected, followed by thorough washing with DI water and ethanol simultaneously until the filtrate became neutral, colorless and odorless. Washing with water and ethanol helps in removing the excess unused oxidant (FeCl_3_) and any pyrrole monomeric/oligomeric units or residual and free TMAO molecules. Thus, the obtained solid was divided into two parts. One part was kept and stored as it is and named as PPy-TMAO, while the other part was subjected to sonication for 12 h continuously followed by thorough washing with ethanol and DI alternatively, and this was then named as MIP. The non-imprinted PPy was also synthesized by following the above steps, but without the addition of TMAO (named as the non-imprinted polymer or NIP). 1.5 mL of PPy-TMAO, MIP, and NIP solutions were then aliquoted in three 2 mL vials and centrifuged at 5000 RPM for 5 min. Water was decanted off from these vials, and 300 μL acetonitrile was added and mixed robustly. This step resulted in the final solutions that were used for the fabrication of electrodes by electrophoretic deposition.

### Fabrication of electrodes for sensing of TMAO

For electrode fabrication, the ITO coated glass plates of dimensions 0.5 × 1.5 cm^2^ were used as electrodes. This required multi-step cleaning of the ITO coated glass substrates by sonicating in EtOH, acetone, and D.I., respectively, for 10 min each. After a thorough cleaning, these ITO sheets were hydrolysed by placing them in a petri dish containing 1:1:5 (volume ratio) solution of H_2_O_2_: NH_3_: H_2_O (hydrogen peroxide: ammonia: water) and by keeping the petri dish at 70°C for 1 h. This was followed by taking the sheets out, rinsing with D.I., and drying at room temperature. Then, the PPy-TMAO, MIP, and NIP electrodes were electrophoretically deposited onto the ITO surface.

**Figure 6:**
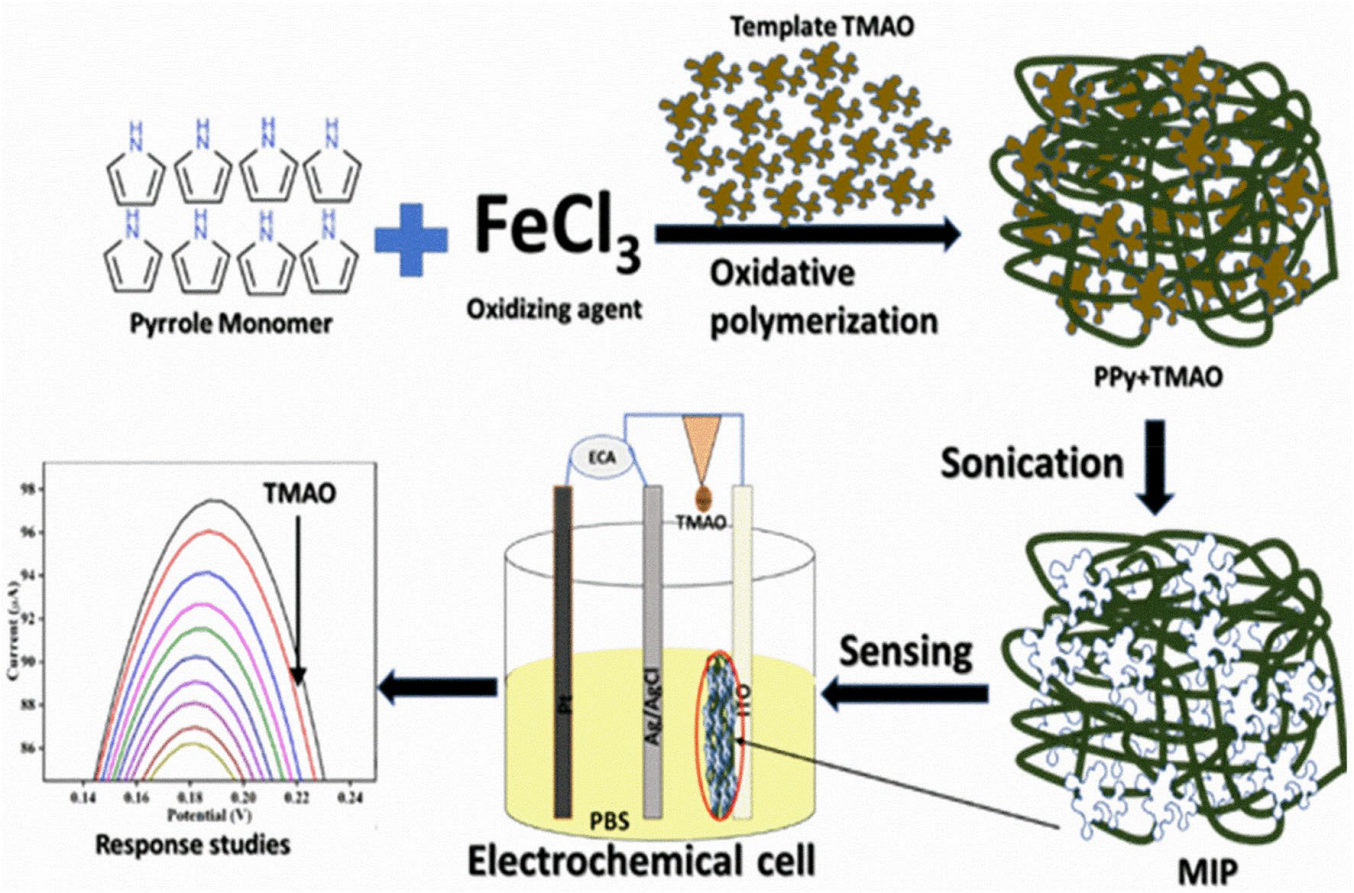
Schematic representation of the MIP synthesis and the fabrication of MIP/ITO electrode towards TMAO detection. The interracial oxidative polymerization was employed for the synthesis of the TMAO imprinted polypyrrole (PPy). MIP/ITO electrode were fabricated by deposition of MIP onto ITO surface electrophoretically, which is further used as a working electrode towards detection of TMAO. The image was created using Microsoft Power Point software, version Microsoft Office 2016.

For electrophoretic deposition of the electrodes, the MIP, NIP, and PPy-TMAO were dispersed in acetonitrile. Mg^++^ ions were used as the catalyst for the deposition. The films were deposited on ITO coated glass plates by applying a voltage of 80 V to the two-electrode cell containing platinum plate as the counter electrode and ITO coated glass plate as the working electrode. The deposition was carried out for 3 min to obtain uniform films with excellent stability of more than six months. The scheme represents the overall synthesis and detection of TMAO based on MIP is shown in Fig. 6.

### Spiked sample preparation

The spike-inmodels were made in the urine. For this 1 mL of clear urine sample was taken and mixed in 4 mL of reconstitution buffer and oscillate for 30 s. To prepare the spiked samples, 20 μL of this pre-treated urine was added to the 20 μL of TMAO of different concentrations. Then, at each concentration, a volume of 20 mL of the spiked sample was added for the response study. This study was carried out in accordance with relevant guidelines and regulations of Jawaharlal Nehru University, New Delhi, India and National Institute of Immunology, New Delhi, India and informed consent was obtained from all volunteers.

### Characterization techniques and TMAO measurement

The structural properties and modification of the MIP, PPy-TMAO, and NIP were analyzed using FT-IR spectroscopy (Perkin Elmer, US). To investigate the topographical and morphological studies of the MIP, PPy-TMAO, and NIP, a Zeiss EVO40 SEM study was carried out. Centrifugation was carried out in an Eppendorf 5424R centrifuge. Further, all the electrochemical investigations [cyclic voltammetry (CV) and differential pulse voltammetry (DPV)] were conducted in a laboratory-made 3-electrode cell with an overall volume of 4 mL using Autolab Galvanostat/Potentiostat electrochemical analyzer (EcoChemie, The Netherlands) connected to a desktop workstation and operated by NOVA and OriginPro 2017 software. Three electrode assembly (silver/silver chloride as a reference electrode against which all potentials were reported; a platinum wire as a counter electrode and MIP/ITO as a working electrode) was used for all the electrochemical measurements. All the measurements were performed atroom temperature and done in phosphate-buffered saline (PBS; 0.2 M, pH 7.4, 0.9% NaCl) containing 5 mM [Fe(CN)_6_]^3−/4−^ as redox species. In CV and DPV, the potential on the working electrode was applied in the range of −0.8 V to 0.8 V for the electro-catalysis experiments. All experimental protocols were approved by ethic committees of Jawaharlal Nehru University, New Delhi and National Institute of Immunology, New Delhi.

## Supplementary Information

Supplementary Information.
