# Supplementary material for: Gut microbiota derived trimethylamine N-oxide (TMAO) detection through molecularly imprinted polymer based sensor
Source: Sci Rep. 2021 Jan 14;11:1338. doi: 10.1038/s41598-020-80122-6 (PMC7809026; doi:10.1038/s41598-020-80122-6)
Supplement: Supplementary file 1 — Supplementary Information. [file 41598_2020_80122_MOESM1_ESM.docx]

Gut microbiota derived trimethylamine N-oxide (TMAO) detection through molecularly imprinted polymer based sensor

G. B. V. S. Lakshmi^1^**^#^**, Amit K. Yadav^1^**^#^**, Neha Mehlawat^2^, Rekha Jalandra^3,4^, Pratima R. Solanki^1^ and Anil Kumar^4^**^*^**

^1^Special Center for Nanoscience, Jawaharlal Nehru University, New Delhi, India

^2^Amity Institute of Applied Sciences, Amity University, Uttar Pradesh, India

^3^Department of Zoology, Maharshi Dayanand University, Rohtak 124001, India

^4^National Institute of Immunology, New Delhi,India

***Corresponding author:** E-mail: [anilk@nii.ac.in](mailto:anilk@nii.ac.in)

^#^Authors contributed equally.

**Supplementary information**

**

**

**Figure S1.** Fourier transform-infrared (FT-IR) spectra of (a) NIP; (b) MIP; (c) PPy-TMAO and (d) TMAO.





**Figure S2.** Scanning electron microscopy (SEM) images of (a) NIP; (b) PPy-TMAO and (c) MIP. (These images were obtained from the SEM system software TESCAN’s Essence™ provided with the instrument.)

The linearity curve among peak current and TMAO concentrations provides the following equation (S11).

**Equations:**

Ipc _(NIP/ITO)_ = [34.16 μA (s/mV) × (scan rate [mV/s])^1/2^] + 36.92 μA, R^2^ = 0.996…............................ (S1)

Ipa_(NIP /ITO)_ = - [26.17 μA (s/mV) × (scan rate [mV/s])^1/2^] – 45.53 μA, R^2^ = 0.995...……………....... (S2)

Ipc_(MIP/ITO)_ = [39.69 μA (s/mV) × (scan rate [mV/s])^1/2^] + 55.65 μA, R^2^ = 0.991................................ (S3)

Ipa_(MIP/ITO)_ = - [30.59 μA (s/mV) × (scan rate [mV/s])^1/2^] – 71.27 μA, R^2^ = 0.986.............................. (S4)

ΔEp (V) _NIP/ITO_ = [0.026 V(s/mV) × (scan rate [mV/s])^1/2^] + 0.912 V, R^2^=0.997…………………. ...(S5)

ΔEp (V) _MIP/ITO_ = [0.021 V(s/mV) × (scan rate [mV/s]) ^1/2^] + 0.088V, R^2^= 0.994............................... (S6)

Ip = (2.69*10^5^) C n^3/2^ D^1/2^ v^1/2^ A ........................................................................................... (S7)

Ae = $\frac{S}{(2.69\times{10}^{5})n^{3}CD^{1/2}}$ .......................................................................................... (S8)

I_p_ = $\frac{n^{2}F^{2}I^{*} A V}{4 R T}$........................................................................................ (S9)

K_s_ = $\frac{mn Fv}{RT}\ldots\ldots\ldots\ldots\ldots\ldots\ldots\ldots\ldots\ldots\ldots\ldots\ldots\ldots\ldots\ldots\ldots\ldots\ldots\ldots\ldots\ldots\ldots\ldots\ldots.$(S10)

Ip = [-0.618 (μA ppm^-1^) × TMAO conc. (ppm) + 94.185 (μA)], R^2^ = 0.981……………… (S11)

LOD= 3 *σ*/k…………………………………………………………………………….…… (S12)
